# Identifying Trauma Patients in Need for Emergency Surgery in the Prehospital Setting: The Prehospital Prediction of In-Hospital Emergency Treatment (PROPHET) Study

**DOI:** 10.3390/jcm12206660

**Published:** 2023-10-20

**Authors:** Stefano Isgrò, Marco Giani, Laura Antolini, Riccardo Giudici, Maria Grazia Valsecchi, Giacomo Bellani, Osvaldo Chiara, Gabriele Bassi, Nicola Latronico, Luca Cabrini, Roberto Fumagalli, Arturo Chieregato, Fabrizio Sammartano, Giuseppe Sechi, Alberto Zoli, Andrea Pagliosa, Alessandra Palo, Oliviero Valoti, Michele Carlucci, Annalisa Benini, Giuseppe Foti

**Affiliations:** 1 Department of Emergency and Intensive Care, Fondazione IRCCS San Gerardo dei Tintori, 20900 Monza, Italy; stefano.isgro@irccs-sangerardo.it (S.I.); marco.giani@unimib.it (M.G.); annalisa.benini@irccs-sangerardo.it (A.B.); 2 Department of Medicine and Surgery, Università degli Studi di Milano-Bicocca, 20126 Monza, Italy; laura.antolini@unimib.it (L.A.); grazia.valsecchi@unimib.it (M.G.V.); roberto.fumagalli@unimib.it (R.F.); 3 Department of Anesthesia and Intensive Care Medicine, Niguarda Hospital, 20162 Milan, Italy; riccardo.giudici@ospedaleniguarda.it (R.G.); gabriele.bassi@ospedaleniguarda.it (G.B.); 4 Department of Anesthesia and Intensive Care, Santa Chiara Regional Hospital, APSS, 38122 Trento, Italy; giacomo.bellani@unitn.it; 5 Centre for Medical Sciences CISMed, University of Trento, 38122 Trento, Italy; 6 Department of Emergency and Trauma Surgery, Niguarda Hospital, 20162 Milan, Italy; osvaldo.chiara@unimi.it; 7 Department of Pathophysiology and Transplantation, Università degli Studi di Milano, 20100 Milan, Italy; 8 Department of Emergency, Spedali Civili University Hospital, 25123 Brescia, Italy; nicola.latronico@unibs.it; 9 General and Neurosurgical Intensive Care Units, Ospedale di Circolo, 21100 Varese, Italy; l.cabrini@asst-settelaghi.it; 10 Department of Biotechnologies and Life Sciences, University of Insubria, ASST Sette Laghi, 21100 Varese, Italy; 11 Department of Anesthesia and Intensive Care Medicine, Neuro Intensive Care, ASST Niguarda, 20162 Milan, Italy; arturo.chieregato@ospedaleniguarda.it; 12 Emergency Department, Emergency and Trauma Surgery, ASST Santi Carlo e Paolo, 20142 Milan, Italy; fabrizio.sammartano@asst-santipaolocarlo.it; 13 Regional Agency of Emergency and Urgency (AREU), 20124 Milan, Italy; g.sechi@areu.lombardia.it (G.S.); a.zoli@areu.lombardia.it (A.Z.); a.pagliosa@areu.lombardia.it (A.P.); 14 Regional Agency of Emergency and Urgency (AREU), 27100 Pavia, Italy; a.palo@areu.lombardia.it; 15 Regional Agency of Emergency and Urgency (AREU), 24121 Bergamo, Italy; o.valoti@areu.lombardia.it; 16 General and Emergency Surgery Department, Ospedale San Raffaele, 20132 Milan, Italy; carlucci.michele@hsr.it

**Keywords:** trauma, triage, emergency surgery, resource allocation, operating room

## Abstract

Prehospital field triage often fails to accurately identify the need for emergent surgical or non-surgical procedures, resulting in inefficient resource utilization and increased costs. This study aimed to analyze prehospital factors associated with the need for emergent procedures (such as surgery or interventional angiography) within 6 h of hospital admission. Additionally, our goal was to develop a prehospital triage tool capable of estimating the likelihood of requiring an emergent procedure following hospital admission. We conducted a retrospective observational study, analyzing both prehospital and in-hospital data obtained from the Lombardy Trauma Registry. We conducted a multivariable logistic regression analysis to identify independent predictors of emergency procedures within the first 6 h from admission. Subsequently, we developed and internally validated a triage score composed of factors associated with the probability of requiring an emergency procedure. The study included a total of 3985 patients, among whom 295 (7.4%) required an emergent procedure within 6 h. Age, penetrating injury, downfall, cardiac arrest, poor neurological status, endotracheal intubation, systolic pressure, diastolic pressure, shock index, respiratory rate and tachycardia were identified as predictors of requiring an emergency procedure. A triage score generated from these predictors showed a good predictive power (AUC of the ROC curve: 0.81) to identify patients requiring an emergent surgical or non-surgical procedure within 6 h from hospital admission. The proposed triage score might contribute to predicting the need for immediate resource availability in trauma patients.

## 1. Introduction

Severely injured patients show better outcomes when treated in high-volume, trauma-specialized hospitals [1,2,3]. This evidence led to the widespread development of structured regional trauma systems [4,5,6] where the most severe patients and complex injuries are addressed to tertiary care facilities classified as level I trauma centers [7]. Prehospital field triage is fundamental to characterize injuries and optimize public health resources, thus allowing the patient to be dispatched to the proper level of care [8,9,10,11].

Even when performed by a physician, prehospital field triage is often imprecise in identifying the need for a level I trauma center [12,13,14], resulting both in under- and over-triage. The precision of prehospital triage further worsens in the prediction of the need for an emergent procedure (surgery, interventional angiography). Less than 15% of patients classified as severely injured subsequently need an urgent (within six hours) or emergent procedure [15]. Prompt availability of an operating room (OR) for surgical emergencies is one of the most critical points [16], bearing the risk of over- and under-triage. Specifically, an overestimation of the need for emergency procedures may lead to interference with surgical activity, inefficient use of resources and higher costs [17,18,19]. On the other hand, unavailability of surgical theaters or interventional radiology may delay urgent treatments, resulting in severe negative outcomes and poor prognosis.

The objective of this retrospective observational study was to identify prehospital predictors of the need for emergency surgical or angiographic procedures (EOR) for trauma patients. Furthermore, we aimed at developing a prehospital triage tool able to predict the need for EOR in the first 6 h after hospital admission.

## 2. Materials and Methods

We performed a retrospective observational study analyzing consecutive records collected in the Regional Lombardy Trauma Registry (see below for details) in the study period between 1 July 2018 (Registry institution) and 31 March 2019. Exclusion criteria were age < 18 years, emergency medical service not involved in patient transportation, patient declared dead on the scene (without interventions) and record missing essential data (see below).

### 2.1. Lombardia Trauma System Organization

Lombardia is a 24,000-square-kilometer region in northern Italy, with about 10 million inhabitants [20], half of which are located in a central highly urbanized zone (Milan Metropolitan Area) and the other half distributed among mountainous and lake areas in the north and flats in the south. EMS is regulated by Regional Agency for Urgency and Emergency (AREU), a free-of-charge regional public health service, and is coordinated by four large EMS call centers, each one covering an orographically homogeneous area (Lakes, Mountains, Metropolitan Area and Flat Area). These directly manage land, air and water vehicles (see Supplementary Table S1 for details of AREU organization).

Hospitals are categorized as highly specialized trauma centers, district trauma centers (level II) and hospitals not specialized in trauma patient care (level III). Since 2012, to guarantee adequate patient volume and, consequently, high quality of care, the EMS operation centers dispatch actual or potential severely injured patients only to the 6 level I trauma centers and to the 12 level II trauma centers.

### 2.2. Trauma Registry

The institution of Lombardy Trauma Registry (LTR) was approved by Ethics Committee Milano Area 2 on 17 July 2018 (record number 569/2018). LTR is a web-based registry, and inclusion criteria of trauma patients are (a) admission to a regional level I or II trauma center and (b) transport by EMS with pre-alert of hospital trauma team or (c) the presence at hospital evaluation of a critical injury in patients without pre-alert of hospital trauma team. Prehospital data are obtained from AREU prehospital digital records (EMMA™ software, Beta 80 Group, Milan, Italy); hospital data are manually inserted in the registry by a data entry team which also assures data quality control.

After being inserted in the LTR, sensitive data are permanently anonymized.

Trauma Registry includes data according to the Utstein template [21]. Records are considered incomplete until, later, each hospital involved in the registry inputs outcome and hospital data and the data entry team verifies and closes the record. For the purpose of this study, only closed records have been included.

### 2.3. Definition of Variables

The registry collected a range of variables, including demographic information such as age and gender, the level of prehospital emergency care activated (basic, advanced or both), time intervals from alert to the scene, time spent on the scene, time and triage code from the scene to the hospital, details about the mechanism of injury (MOI) and its intentionality. Additionally, it recorded prehospital events such as endotracheal intubation, out-of-hospital cardiac arrest (OHCA) and fatal outcomes, as well as injury severity scores (injury severity score—ISS), hospital procedures such as damage control surgery and interventional radiology and outcomes including hospitalization, length of stay, admission to the intensive care unit (ICU), duration of invasive ventilation and patient status at the time of hospital discharge. Notably, prehospital vital signs were only collected for patients who did not experience cardiac arrest. Shock index [22] was then calculated as heart rate divided by the systolic blood pressure. In cases where only the basic trauma care provider was involved, the neurological evaluation was limited to the alert/verbal/pain/unresponsive (AVPU) scale. It should be noted that, in Lombardy, basic life support units are comprised of two to three non-professional volunteer individuals. However, when advanced trauma teams were engaged, both the AVPU scale and the Glasgow coma scale (GCS) were recorded, although only the GCS was taken into consideration.

We defined as requiring emergency operating room (EOR) those patients who underwent surgical or angiographic procedure within 6 h from ED arrival (thoracotomy, laparotomy, craniotomy, preperitoneal packing, intracranial pressure probe positioning, arterial revascularization, angiographic embolization) or patients who arrived alive in the ED but who died before any possible surgical treatment could be accomplished. We will refer to the latter subgroup as “potential EOR”.

This study was conducted and reported based on the Strengthening the Reporting of Observational Studies in Epidemiology (STROBE) guidelines.

### 2.4. Statistical Analysis

Continuous data were described by mean and standard deviation or by median (25th–75th percentiles), as appropriate. Categorical data were described by count and percentages. Comparison across groups defined by the need or no need for EOR (EOR group versus noEOR group) was obtained by Student’s T test and chi-square test.

The probability of the need for EOR was related to possible explanatory factors by univariate logistic regression models. Prehospital vital signs were included by 3-knots restricted cubic splines to enlighten reasonable categorizations (i.e., U-shaped relationships). A multivariable logistic regression model was then estimated including prehospital vital signs as interactions in the absence of cardiac arrest. Model results were used to develop a tool (Excel spreadsheet) that allows for the calculation of the predicted probability of needing EOR (prediction of EOR).

The model’s predictive performance was assessed by the area under the ROC curve.

To assess the robustness of the model’s predictive performance, the original sample was randomly split into a training set (75% random selection of the whole sample) and testing set (the remaining 25% of the whole sample). The model was re-estimated on the training set, and the predictive performance was assessed on the testing set for internal validation by the area under the ROC curve and the likelihood ratio test based on the calibration belt method. Data were analyzed with STATA 16 ^®^ (StataCorp LLC, College Station, TX, USA).

## 3. Results

**Figure 1:**
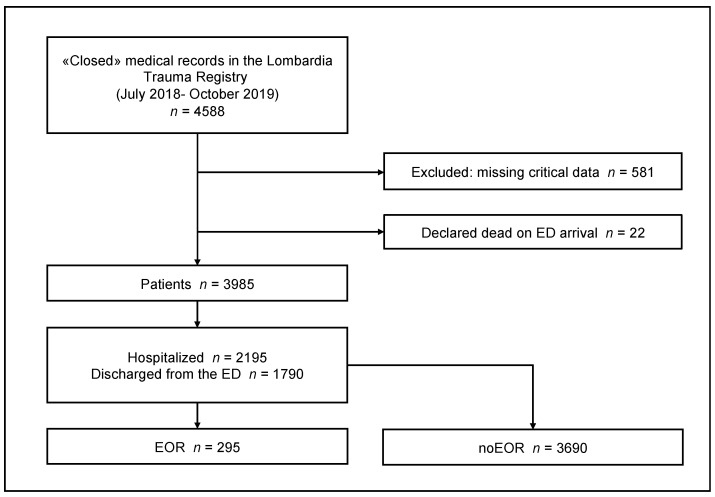
Study flowchart. ED, emergency department; EOR, emergency operating room or interventional radiology.

A total of 4588 patients were included in the Trauma Registry in the study period. In total, 603 patients were excluded (581 due to missing critical data and 22 were declared dead on ED arrival). The remaining 3985 patients were enrolled in the study (see study flowchart, Figure 1).

Of these, 295 (7.4%) patients were included in the EOR group: 245 of them underwent an emergency procedure (EOR), while the remaining 50 patients died in the ED before any possible surgical intervention (potential EOR). Overall, 192 (4.8%) patients died. The remaining 3690 did not require EOR: 1790 of them were discharged home from the ED, whereas 1900 were hospitalized. Out of the 245 patients who received an emergency procedure, 96 (39%) underwent neurosurgery (craniotomy and/or ICP catheter positioning), 83 (34%) emergent angiographic embolization, 59 (24%) exploratory laparotomy, 16 (6.5%) preperitoneal packing and 11 (4.5%) thoracotomies.

The median time spent on the scene was 28 (22–37) min, while time spent from EMS arrival on the scene to hospital admission was 42 (33–52) min.

Table 1 shows the characteristics of the study population. Compared to the noEOR group, the EOR patients were older, showed poorer neurological scores, had lower arterial pressure, higher heart rate, higher shock index (average of 0.81 ± 0.43 vs. 0.67 ± 0.2, *p* < 0.001) and a higher injury score (average ISS of 27.1 ± 17 vs. 6.5 ± 8.7, *p* < 0.001). Penetrating trauma was more common in the EOR group (6.1% vs. 3.0%, *p*-value = 0.003), and mechanisms of injury differed significantly between groups (*p* < 0.001). Specifically, fall from heights was the most common cause of trauma in the noEOR group, whereas motorcycle accidents were the most common mechanism of injury in the EOR group. OR patients were more frequently intubated (44.8% vs. 3.9%, *p*-value < 0.001) and admitted to the ICU (74% vs. 11%, *p*-value < 0.001). Hospital mortality of the EOR group was 36.6% vs. 2.3% in the noEOR group (*p*-value < 0.001). In patients without OHCA, the risk of EOR as a function of systolic blood pressure, diastolic blood pressure and heart rate showed a U-shaped behavior (see Figure S1). These variables were included in the logistic models as categorical variables according to the following cut-points: 90 and 180 mmHg for systolic blood pressure, 50 and 90 mmHg for diastolic blood pressure, 60, 100 and 120 beats per minute for heart rate, 15 and 30 breaths per minute for respiratory rate and 0.7 and 1.3 for the shock index.

At the univariate analysis, factors associated with increased probability for EOR were age, penetrating injury, downfall, cardiac arrest, poor neurological status, endotracheal intubation, systolic pressure, diastolic pressure, shock index, respiratory rate and tachycardia (see Table 2). At the multivariable logistic regression analysis, age, penetrating injury, downfall, cardiac arrest, poor neurological status and endotracheal intubation were independently associated with the need for EOR (Table 2). The prediction of EOR depending on the patient characteristics was calculated as follows:

exp (total score)/[1 + exp (total score)]

where the total score is obtained by summing up the scores provided in Supplementary Table S2 to the constant value (−3.71). A spreadsheet for the automatic calculation of the prediction of EOR is provided as Supplementary Materials.

**Figure 2:**
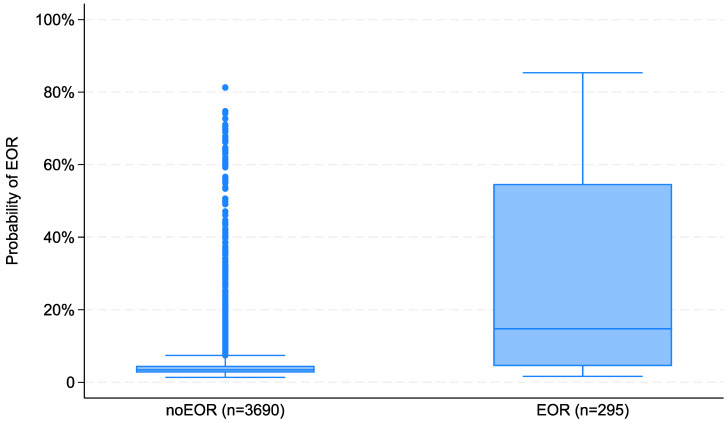
Boxplot of the prediction of the need of emergency operating room (EOR) in the original sample (n = 3985).

The distribution of the prediction of EOR in the original sample in subgroups defined by the need of EOR is displayed in Figure 2.

**Figure 3:**
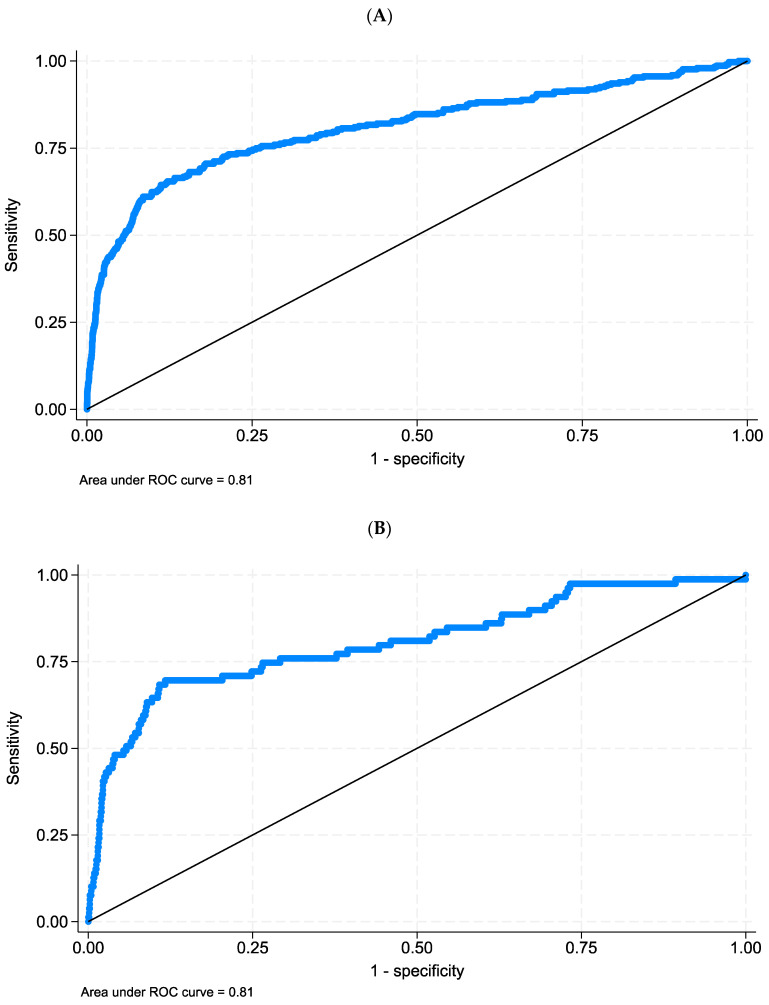
ROC curves. (**A**) Prediction of the need for EOR calculated on original dataset (n = 3985). (**B**) ROC curve of the prediction of the need for EOR obtained from the training set and calculated on the testing set (n = 982).

The area under the ROC curve defined by the prediction of EOR is 0.81 (95% CI 0.78 to 0.84) (Figure 3A). The likelihood ratio test on calibration yielded a *p*-value of 0.126. This result was expected because calibration is assessed on the development data.

The multivariable logistic regression model was re-estimated on a random selection of 75% of the sample (*n* = 3003, training set). The risk predictions were then applied on the remaining 25% of the sample (*n* = 982, testing set). The area under the ROC curve calculated on the testing set was 0.81 (95% CI 0.75 to 0.87) (Figure 3B). The likelihood ratio test on calibration yielded a *p*-value of 0.297. This suggests that the predictions of the model developed in the training data set do not significantly deviate from the observed rate, even in the testing set.

## 4. Discussion

In the present study, we analyzed prehospital factors associated with the need for emergency procedures (i.e., surgical or radiological) within 6 h from ED admission. Several independent predictors were identified, and a triage score was built on these variables, allowing for the calculation of the predicted probability of EOR (ROC curve AUC of 0.81 both in the whole population and in the testing cohort).

Several studies have explored the impact of prehospital factors on various hospital outcomes.

In 2017, Thompson et al. [5] studied a predictive prehospital triage model with hospital mortality as the primary outcome, analyzing 1033 EMS patient records. Variables independently associated with unfavorable outcome were GCS, out of range respiratory rate, hypotension, age, transport time, crew skill mix and triage practices. The authors concluded that GCS, respiratory rate and age were the best predictors for outcome, especially when compared with factors depending on EMS system organization.

Van Rein et al. [23] chose ISS ≥ 15 as an outcome and analyzed field variables collected from regional EMS reports to compute a triage model. The final model included eight predictors: age, systolic blood pressure, GCS, penetrating injury, head/thorax injury and multiregional injury. The prediction model was associated with an over-triage (i.e., overestimation of ISS) rate of 50% and an under-triage rate of 11.2%.

Few studies have focused on the need for urgent in-hospital intervention as an outcome. In 2005, Holcomb et al. [24] identified hypotension and motor GCS impairment as the strongest field predictors for in-hospital intervention in the first 24 h. The authors reported that 21% of patients without alterations of physiological parameters in the field required a lifesaving hospital procedure. The study population was relatively small (216 patients), and a high rate of hospital procedure (37%) was reported. In 2006, Steele et al. [15] retrospectively investigated the field triage criteria among those indicated by the American College of Surgeons, which mandates a surgeon’s presence in the ED upon trauma arrival, and identified the patients subsequently treated by a general surgeon within 1 h (confirmed systolic blood pressure < 90 mmHg, respiratory compromise/airway obstruction or intubation, inter-hospital transfer of patients receiving blood transfusion to maintain vital signs, torso gunshot wounds, GCS < 8 due to trauma). The adult study population was 5978; Steele reported that 3% of emergency intervention was performed by the trauma surgeon; factors strongly associated with the need for intervention were torso gunshot wounds, confirmed hypotension and ongoing blood transfusion. In 2012, Lin et al. [12] prospectively studied 601 trauma patients with the aim of identifying the prehospital factors predictive for emergent hospital interventions. Trauma alert criteria were based on three clinical items (airway, consciousness and circulation), two anatomical items (presence of long bone fractures, amputation), mechanism of injury (penetrating) and the presence of a high index of suspicion as indicated by the paramedics in the field. EOR was needed in 6.5% of patients.

Other studies included the need for emergency intervention in composite outcomes. Van Haren et al. [25] tested a composite outcome of hospital intubation, transfusion and emergency intervention by combining oxygen saturation below 95%, heart rate above 100 and systolic pressure below 90 mmHg and reported a specificity of 78% and sensitivity of 14% for penetrating injuries and, respectively, 74% and 49% for blunt injuries. In 2020, Newgard et al. published a meta-analysis about hemodynamic predictors of resources use, injury and mortality, demonstrating low sensitivity and high specificity for all the predictors included, without evidence of any parameter being superior when compared to another [26].

In the present study, we restricted our analysis to EOR within 6 h after admission to the ED. The aim of this study was, in fact, to find a tool to optimize the allocation of trauma patients within the trauma system and to decrease the suboptimal utilization of higher-level trauma centers’ resources, such as operating theaters. Cardiac arrest, endotracheal intubation, hypotension, penetrating injury and falling as mechanisms of injury and neurological impairment were the strongest predictors for EOR need. Respiratory triage parameters generally show low sensitivity and high specificity [27]; the association of prehospital intubation for respiratory compromise or obstruction and the need for EOR is debated, as clinical indications to intubate are wide, and many of these patients did not subsequently show evidence of ongoing hemorrhage or the need for neurosurgical intervention in the ED. In previous studies [19,28], only 45% of patients intubated in the field needed EOR. These results are similar to those reported by Ciesla et al. [19]. Nevertheless, in our model, intubation was strongly predictive of the need for EOR. Oxygen peripheral saturation was not associated with EOR in univariate analysis while respiratory rate was predictive for EOR in univariate analysis but lost predictive power in the multivariable logistic regression. HR was not predictive when combined in multivariable analysis, both if greater than 100 and of 120 bpm. This result is consistent with other evidence of a scarce diagnostic value of HR as compared to other circulatory parameters [26]. Our study showed a significant over-precautionary activation of the operating rooms in trauma center hospitals, with an effective need for EOR of 7.4%, which was similar to that reported by Lin et al. [12]. Our score showed a good predictive power, with an AUC of 0.81 and 0.81 in the whole sample and testing set, respectively.

The main strength of this study relies on the very large study population, which allowed the identification of several independent predictors of needing EOR. This represents a novelty in the current literature, as no study to date has focused on the predictors of emergency need for a surgical or angiographic procedure (i.e., within 6 h) in trauma patients. Moreover, we developed a score to predict the probability of emergency resource use, which might contribute to optimizing patient allocation.

The present study also has some limitations. First, during the study period, the Trauma Registry data collection area was progressively expanded to cover the whole region. For this reason, characteristics and transport times may have varied during the study period. Moreover, data availability is limited by their presence in the registry itself. Potential informative data such as pupil diameter, external hemorrhage, pelvic binder utilization, confirmation of hypotension by a second set of hemodynamic parameters or large resuscitation volumes were not included in the records. Second, due to the prevalence of patients in the metropolitan district, the median transportation time was short (13 min), and most patients reached the hospital within the “golden hour”. Therefore, our results might not be generalizable to other settings and territories. Third, we used 6 h after admission to the emergency department as a time cutoff for defining an emergency procedure. This cutoff was chosen arbitrarily, as we were not able to find any more valid cutoff in previous literature. Last, we cannot rule out that patients who were excluded due to missing data were more severely injured than those included in our population, as missing information may have been directly related to the workload of the emergency crew in the field.

## 5. Conclusions

In a large cohort of injured patients, we identified several predictors of in-hospital need for emergency procedures. A prehospital score built to predict the need for emergency intervention within 6 h from hospital admission provides the probability of undergoing an emergent procedure with good accuracy. Further studies may focus on the trend of vitals and other clinical parameters over time, which may have a higher predictive value compared to the static parameters which were included in our work.

## Figures and Tables

**Table 1:** Study population stratified by utilization of emergency operating room (EOR) within the first 6 h.

	Overall (n = 3985)	noEOR Group (n = 3690)	EOR Group (n = 295)	*p*-Value
Demographic variables				
Age (years)	48.7 ± 23.4	48.4 ± 23.2	52.9 ± 25.4	0.014
Gender (male)	2830 (71.0)	2608 (70.7)	222 (75.3)	0.095
Mechanism of trauma				
Penetrating trauma	128 (3.2)	110 (3)	18 (6.1)	0.003
Injury mechanics				<0.001
Weapon	138 (3.5)	118 (3.2)	20 (6.8)	
Motor vehicle	717 (18)	676 (18.3)	41 (13.9)	
Downfall (high altitude)	1043 (26.2)	993 (26.9)	50 (17)	
Bicycle	355 (8.9)	333 (9)	22 (7.5)	
Motorcycle	878 (22)	808 (21.9)	70 (23.7)	
Pedestrian	442 (11.1)	409 (11.1)	33 (11.2)	
Fall (i.e., horse, bed, chair, stairs)	315 (7.9)	263 (7.1)	52 (17.6)	
Crushing	97 (2.4)	90 (2.4)	7 (2.4)	
Clinical parameters				
Neurological scores				<0.001
AVPU = ALERT	1234 (31)	1201 (32.6)	33 (11.2)	
AVPU = VERBAL	50 (1.3)	44 (1.2)	6 (2)	
AVPU = PAIN	19 (0.5)	18 (0.5)	1 (0.3)	
GCS >13	2317 (58.1)	2211 (59.9)	106 (35.9)	
GCS = 9–13	151 (3.8)	113 (3.1)	38 (12.9)	
GCS = 3–8 or AVPU = UNRESPONSIVE	214 (5.4)	103 (2.8)	111 (37.6)	
				
Systolic blood pressure *	135 ± 26.7	135.5 ± 26	127.6 ± 35.1	<0.001
Diastolic blood pressure *	79.9 ± 15.1	80.2 ± 14.8	75.1 ± 18.9	<0.001
Heart rate *	88 ± 18.6	87.7 ± 18.2	92.9 ± 23.5	<0.001
Respiratory rate *	15.9 ± 3.8	16 ± 3.7	15.6 ± 5.5	0.149
Shock index	0.68 ± 0.22	0.67 ± 0.2	0.81 ± 0.43	<0.001
Other parameters				
Injury severity score	8 ± 11	6.5 ± 8.7	27.1 ± 17	<0.001
Endotracheal intubation	276 (6.9%)	144 (3.9%)	132 (44.8%)	<0.001
Outcomes				
ICU admission	624 (15.7%)	406 (11%)	218 (74%)	<0.001
Hospital mortality	193 (4.8%)	85 (2.3%)	108 (36.6%)	<0.001

Data are presented as mean ± standard deviation or count and percentages. * Data from 3915 patients.

**Table 2:** Univariate and multivariable logistic regression model on the risk of requiring an emergency operating room (EOR).

	Univariate Analysis		Multivariable Analysis	
Variable	Odd Ratio (95% CI)	*p*-Value	Odd Ratio (95% CI)	*p*-Value
Age (per year increase)	1.01 (1–1.01)	0.001	1.01 (1–1.01)	0.028
Penetrating trauma (ref. blunt)	2.11 (1.27–3.53)	0.004	2 (1.1–3.66)	0.024
Injury from fall (ref. No., i.e., other mechanisms)	2.79 (2.02–3.86)	<0.001	2.03 (1.37–3)	<0.0001
Neurological status (ref. GCS > 13)				
AVPU = ALERT	0.57 (0.39–0.85)	0.006	0.63 (0.42–0.96)	0.030
AVPU = VERBAL	2.84 (1.19–6.82)	0.019	2.4 (0.94–6.13)	0.068
AVPU = PAIN	1.16 (0.15–8.76)	0.886	0.92 (0.12–7.17)	0.938
GCS = 9–13	7.01 (4.63–10.63)	<0.001	4.3 (2.67–6.92)	<0.0001
GCS = 3–8 or AVPU = UNRESPONSIVE	22.48 (16.14–31.31)	<0.001	5.7 (3.26–9.99)	<0.0001
Cardiac arrest	28.22 (16.95–46.99)	<0.001	3.08 (1.6–5.94)	0.001
Systolic blood pressure, mmHg (ref. 90–180)				
<90	6.15 (3.93–9.63)	<0.001	2.14 (0.96–4.78)	0.064
>180	1.59 (0.93–2.7)	0.089	1.1 (0.56–2.19)	0.777
Diastolic blood pressure, mmHg (ref. 50–90)				
<50	4.34 (2.57–7.33)	<0.001	0.97 (0.37–2.55)	0.948
>90	0.69 (0.51–0.92)	0.011	0.84 (0.58–1.22)	0.359
Heart rate, bpm (ref. 60–100)				
<60	1.69 (0.92–3.13)	0.092	1.14 (0.56–2.35)	0.713
100–120	1.65 (1.19–2.27)	0.003	1.14 (0.75–1.72)	0.535
>120	2.16 (1.28–3.64)	0.004	1.38 (0.71–2.68)	0.348
Respiratory rate, breaths/min (ref. < 15)				
15–29	0.66 (0.49–0.88)	0.005	0.9 (0.66–1.24)	0.525
≥30	4.1 (1.99–8.44)	<0.001	1.99 (0.85–4.68)	0.113
Shock index (ref. < 0.7)				
0.7–1.3	1.58 (1.21–2.07)	0.001	1.37 (0.95–1.97)	0.089
≥1.3	7.11 (4.01–12.61)	<0.001	1.05 (0.4–2.73)	0.924
Endotracheal intubation (ref. No.)	19.94 (15.02–26.48)	<0.001	2.86 (1.73–4.73)	<0.0001

CI, confidence interval.

## Data Availability

Data can be obtained from the corresponding author, upon reasonable request, after approval from the local Ethics Committee.

## Supplementary Materials

The following supporting information can be downloaded at: https://www.mdpi.com/article/10.3390/jcm12206660/s1, Figure S1: Relationship between heart rate (Panel A), systolic blood pressure (Panel B) and diastolic blood pressure (Panel C) with the probability of requiring emergency operating room (EOR); Table S1: Emergency medical service vehicles characteristics; Table S2. Coefficients used to calculate the predicted risk of requiring emergency operating room (EOR); Prophet Score Calculator.xls.
