# Supplementary material for: Identifying Trauma Patients in Need for Emergency Surgery in the Prehospital Setting: The Prehospital Prediction of In-Hospital Emergency Treatment (PROPHET) Study
Source: J Clin Med. 2023 Oct 20;12(20):6660. doi: 10.3390/jcm12206660 (PMC10607301; doi:10.3390/jcm12206660)
Supplement: Supplementary file 1 [file jcm-12-06660-s001.zip › Supplement PROPHET 2.0.pdf]

Supplementary Material

Identifying trauma patients in need for emergency surgery in the prehospital setting: The PRehOspital Prediction of in Hospital Emergency Treatment (PROPHET) Study

Supplementary Results

A

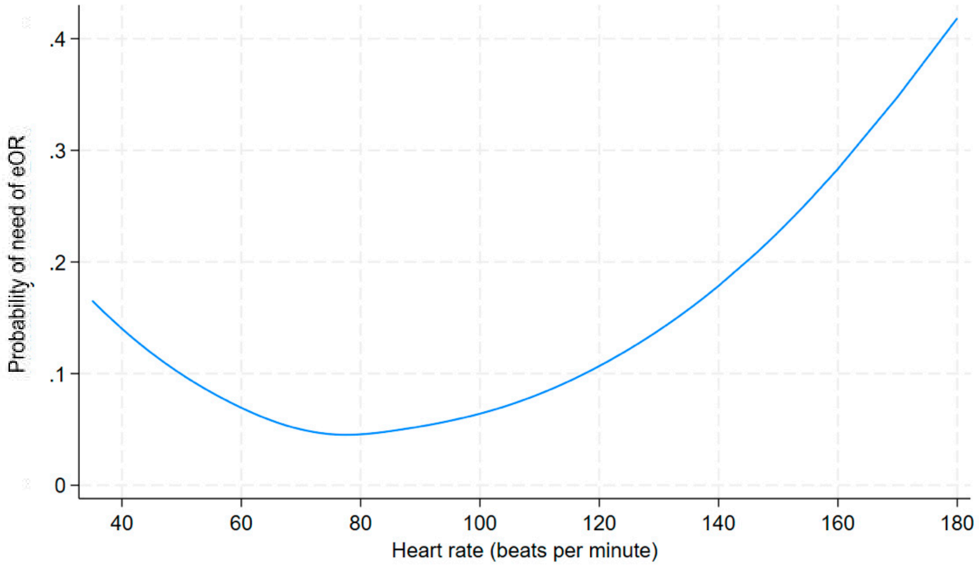

B

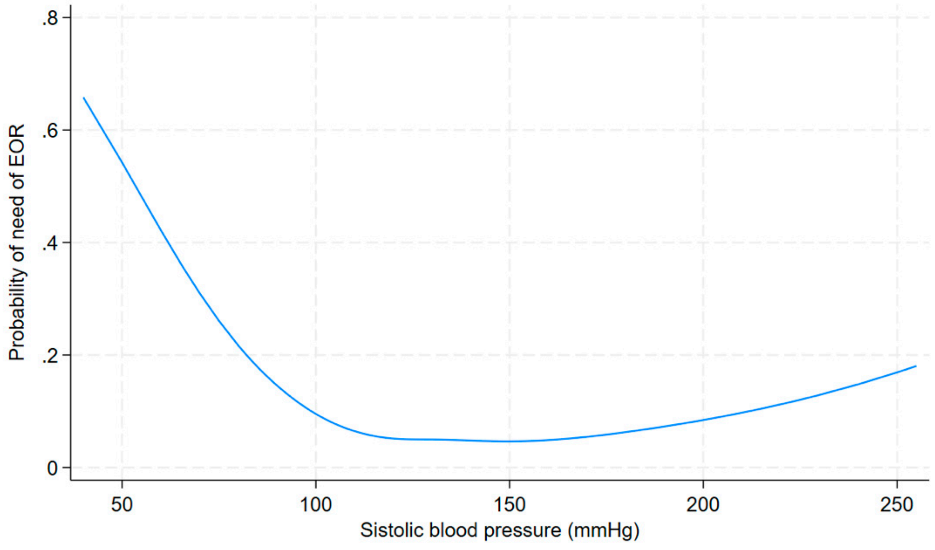

C

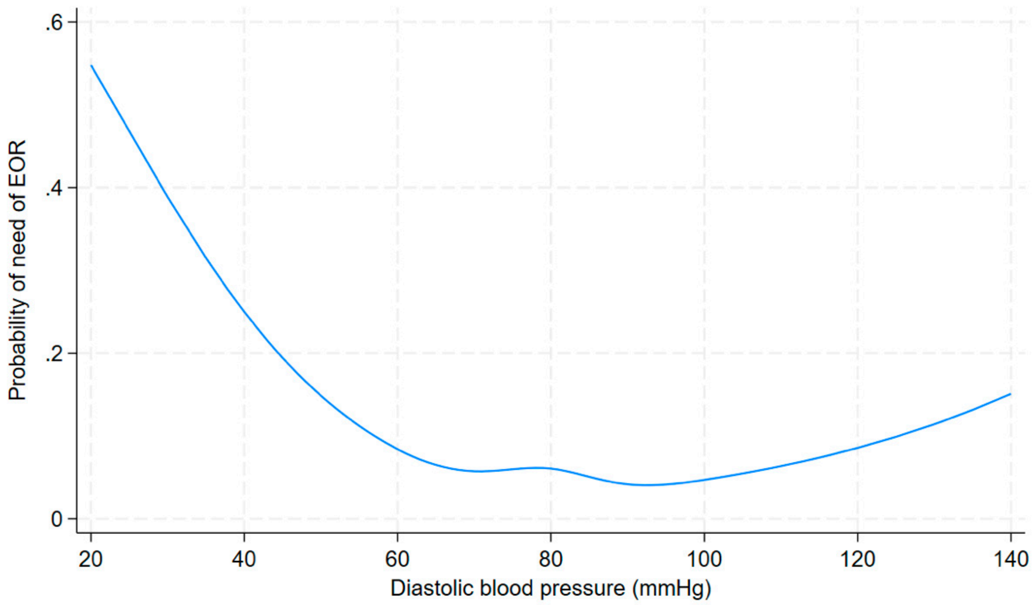

Figure S1. Relationship between heart rate (Panel A), systolic blood pressure (Panel B) and diastolic blood pressure (Panel C) with the probability of requiring emergency operating room (EOR).

		Functions	Training	Team
Land vehicles	Ambulance	First Response transportation	BTS	Up to 4 Basic Life Support trained responders (volunteers, professional rescuers)
	Car	Advanced response	BTS<br>ATS	Driver – RN
	Car	Advanced response	BTS<br>ATS	Driver - RN - physician
Water vehicles	Motorboat	Advanced response - transportation	BTS<br>ATS	Driver - RN - physician
Air vehicles	Helicopter	Advanced response - transportation	BTS<br>ATS	Pilot – flight technician – RN - physician

Table S1: Emergency medical service vehicles characteristics. RN: registered nurse; ATS, advanced trauma support; BTS, basic trauma support.

Table S2. Coefficients used to calculate the predicted risk of requiring emergency operating room (EOR). Coefficients were derived from the multivariable logistic regression evaluating the independent association of the covariates with the risk of EOR.

Variable	Coefficient
**Age (years)**	0.007
**Penetrating trauma**	0.695
**Injury from fall**	0.706
**Neurological status (ref. GCS>13)**	
GCS = 9-13	1.459
AVPU = ALERT	-0.456
AVPU = VERBAL	0.875
AVPU = PAIN	-0.081
GCS = 3-8 or AVPU = UNRESPONSIVE	1.741
**Cardiac Arrest**	1.127
**Systolic blood pressure (ref 90-180 mmHg)***	
<90 mmHg	0.761
>180 mmHg	0.099
**Diastolic blood pressure (ref. 50-90 mmHg)***	
<50 mmHg	-0.033
>90 mmHg	-0.172
**Heart rate (ref. 60-100/min)***	
<60	0.135
100-120	0.131
>120	0.319

**Respiratory rate (ref <15/min)***	
15-29	-0.103
>=30	0.690
**Shock index (ref. <0.7)**	
0.7-1.3	0.316
>1.3	0.046
**Endotracheal intubation (ref. No)**	1.052

* to be considered only on patients who did not develop cardiac arrest
